# In Vitro Evaluation of Enzymatically Transformed Alfalfa Saponins on Methane Reduction, Rumen Microbes and Metabolomics in Goats

**DOI:** 10.3390/ani15111516

**Published:** 2025-05-22

**Authors:** Ran Zhang, Xinran Bao, Xingqi Shi, Shixuan Jin, Ying Meng, Zhiwei Li, Zhumei Du, Xuebing Yan

**Affiliations:** College of Animal Science and Technology, Yangzhou University, Yangzhou 225009, China; dx120210163@stu.yzu.edu.cn (R.Z.);

**Keywords:** feed additive, β-glucosidase, in vitro, ruminant nutrition, methane mitigation, metabolomics

## Abstract

Alfalfa (*Medicago sativa* L.) saponins (AS), primarily pentacyclic triterpenoids, may reduce methane emissions from goats (*Capra hircus* L.). This study evaluated the methane-suppressing potential of *Aspergillus niger* β-glucosidase-modified AS using in vitro rumen fermentation (0.10 mg/mL inoculum, 24 h incubation, gas chromatography detection). Among the 21 alfalfa cultivars, Pegasis (fall dormancy 9) exhibited the highest antioxidant efficacy (half maximum effective concentration 2.13 mg/mL) and the lowest ferric-reducing activity (0.32 μM Fe^2+^/g) (*p* < 0.05). Fresh/silage AS reduced methane proportions to 4.50–5.21% of total gas, while enzymatic biotransformation further decreased it to 3.34–3.48% (*p* < 0.05). Methanogen abundance declined by 20.10–44.93%, and general anaerobic fungi declined by 34.22–44.66% compared to untreated AS (*p* < 0.05). Metabolomics linked methane suppression to six pathways, including zeatin biosynthesis (via nucleotide metabolites accumulation) and prolactin signaling pathway (via bioactive molecules downregulation), suggesting impaired methanogen energy metabolism and hydrogen flux redirection as mechanisms. Enzymatic AS also enhanced volatile fatty acid production, indicating improved fiber digestion. These in vitro findings demonstrate that enzyme-treated AS modulates rumen fermentation through dual methane mitigation and nutrient utilization enhancement, offering a sustainable feed additive strategy for livestock.

## Simple Summary

Alfalfa, a common livestock feed, contains natural compounds known as saponins, which may help reduce methane emissions from goats, a major contributor to climate change. This study investigated the use of a food-safe enzyme derived from *Aspergillus niger* to enhance the activity of alfalfa saponins. Saponins from 21 alfalfa cultivars were tested, showing stronger antioxidant activity after silage fermentation. Adding a small amount (0.10 mg/mL) of saponins to goat rumen fluid during in vitro fermentation reduced methane production and altered the rumen microbes linked to methane release. When the enzyme-treated saponins were used, methane levels dropped further, while beneficial microbes and nutrients linked to animal growth increased. The enzymatically enhanced saponins also influenced key metabolic pathways in the goats’ digestive processes, suggesting a dual benefit: lowering greenhouse gas emissions and improving feed efficiency. This eco-friendly approach demonstrates how enzymatically transformed alfalfa saponins could make livestock farming more sustainable by mitigating methane emissions.

## 1. Introduction

Alfalfa (*Medicago sativa* L.), a globally important forage crop, serves as a rich source of proteins, carbohydrates, and bioactive phytochemicals. Among these compounds, alfalfa saponins (AS)—primarily pentacyclic triterpenoids, such as bayogenin, hederagenin, and soyasapogenol glycosides, have garnered attention for their cholesterol-lowering, immunostimulant, and antioxidant properties [1]. Notably, AS demonstrate exceptional potential as natural feed additives for ruminants, with methane mitigation capabilities [2]. However, the practical application of AS is hindered by their bitter taste (primarily attributed to zanhic acid, a dominant triterpenoid acid in alfalfa with hydroxyl and carboxyl groups that activate mammalian bitter taste receptors) and variable bioactivity across cultivars [3]. These challenges necessitate processing strategies that simultaneously improve saponin palatability and enhance their methane-suppressing efficacy. Recent advances in forage processing reveal that ensiling not only doubles the total concentration of saponins, but triggers structural transformations [4]. The AS directly inhibit hydrogenotrophic methanogens by disrupting key enzymes (e.g., methyl-CoM reductase) and indirectly reduce hydrogen availability through microbial community shifts [5], highlighting the critical role of feed processing technologies in unlocking the nutritional value of phytochemicals.

The imperative to develop sustainable livestock production systems has intensified the search for dietary strategies that concurrently address methane emissions and feed efficiency. Ruminant methane, contributing 30–40% of global agricultural greenhouse gas emissions [5], represents both an environmental liability (298 × CO_2_-equivalent warming potential) and a 2–12% net energy loss for the host animal [6]. While AS is known to suppress methanogens and redirect hydrogen toward propionate production [7], their efficacy is limited by the steric hindrance caused by β-1,4-glycosidic side chains. This limitation underscores the need for enzymatic bioprocessing, a frontier in feed additive innovation, to amplify the bioactivity of plant-derived phytochemicals.

β-Glucosidases from *Aspergillus niger*, optimized for industrial-scale production [8], offer a viable solution. β-Glucosidase from *A. niger* effectively breaks down glycosidic bonds and remains stable at 40 to 55 °C and pH 4.5 to 6.0 [9], as evidenced by its successful application in ginsenoside bioactivation [10]. Despite this promise, the use of β-glucosidase to enhance AS’ methane-suppressing potential remains unexplored—a knowledge gap critical to advancing phytochemical-based feed technologies. Furthermore, the existing studies predominantly focus on fresh AS, neglecting the combined effects of ensiling and enzymatic treatment on saponin transformation.

These research gaps are addressed through a systematic study into the following: (1) cultivar selection: screening the AS of 21 alfalfa cultivars for antioxidant capacity linked to fall dormancy (FD)—a trait ranking alfalfa’s autumn growth cessation (1 = complete dormancy; 11 = no dormancy) that shapes secondary metabolites (e.g., saponins) via stress-response pathways (low-FD cultivars upregulate defense compounds for winter survival) [3]; (2) feed processing innovation: employing *A. niger* β-glucosidase to optimize AS bioactivity in both fresh and ensiled materials; (3) sustainable methane mitigation: establishing the dose–response relationship between enzymatic AS and in vitro methane reduction while evaluating trade-offs in rumen fermentation parameters; (4) mechanistic insights: integrating microbes–metabolome analyses to elucidate how enzymatic AS remodel microbes hydrogen metabolism and nutrient utilization pathways.

This study provides the first evidence that enzymatic biotransformation targeting β-1,4-glycosidic side chains synergizes with ensiling to maximize AS’ methane mitigation potential. The β-1,4-glycosidic side chains of AS act as steric hindrance moieties, limiting interactions with methanogen targets, but enzymatic cleavage of these chains enhances the AS bioavailability by exposing its triterpenoid core [11]. The findings guide the creation of smart forage systems to meet global demands for phytochemical-based ruminant nutrition solutions.

## 2. Materials and Methods

### 2.1. Antioxidant Activity of AS

#### 2.1.1. Silage Preparation

First-cut alfalfa was harvested at the early bloom stage (10% flowering) from experimental plots at Yangzhou University (Yangzhou, China) on 28 April 2023. FD ratings follow the US Department of Agriculture classification system, where lower FD values indicate stronger winter dormancy (FD4: semi-dormant; FD10: non-dormant) [3] (Table 1). Fresh alfalfa was dried and crushed and then used to extract fresh AS. After wilting alfalfa for 6 h (moisture content reduced to 65%), the additive containing lactic acid bacteria was applied at a rate of 1 × 10^11^ CFU per ton of fresh weight. The inoculant consisted of a single-strain *Lactiplantibacillus plantarum* CAU-a214 (dominant species > 99%), achieving a final concentration of 1 × 10^6^ CFU/g wet silage after thorough mixing. The material was packed into plastic silos, compacted to 700 kg/m^3^, sealed, and stored at room temperature (25 ± 2 °C). After 60 days of ensiling, the silos were opened, and the contents were dried and crushed before being used to extract silage AS.

#### 2.1.2. Chemical Analysis

The dry matter (DM) content of fresh and ensiled alfalfa samples was assessed according to the AOAC method 934.01. Crude protein (CP) content was measured with a Hanon K9860 Automatic Kjeldahl Nitrogen Analyzer (Jinan Hanon Instruments Co., Ltd., Jinan, China) (AOAC 976.05). Ether extract (EE) content was measured with a Shanghai Xianjian SZC-101 Automatic Soxhlet Extractor (Shanghai Xianjian Instruments Co., Ltd., Shanghai, China) (AOAC 920.39). Neutral detergent fiber (NDF) and acid detergent fiber (ADF) were performed using a Jinan Languang F800 Fiber Analyzer (Jinan Languang Electromechanical Technology Co., Ltd., Jinan, China) (AOAC 2002.04; 973.18) [12]. Ammonia-N levels were quantified using a Hanon K9860 Automatic Kjeldahl Nitrogen Analyzer (AOAC 941.04), pH was measured with a PHS-3E digital pH meter (Shanghai Yidian Scientific Instruments Co., Ltd., Shanghai, China). Lactic acid, acetic acid, and butyric acid content was determined using a P270 HPLC System (Dalian Elite Analytical Instruments Co., Ltd., Dalian, China) in the alfalfa silage (*n* = 4 biological replicates) [4].

#### 2.1.3. Saponin Extraction

An accelerated solvent extraction system (ASE 200, Dionex Corp., Sunnyvale, CA, USA) was used to prepare the extracts [4]. Samples (0.10 g) were mixed with diatomaceous earth (ASE Prep DE) and extracted with 80% MeOH. The extraction was carried out at 10 MPa and 40 °C. Dried samples were dissolved in methanol and then purified with solid-phase extraction. Waters Sep-Pak Classic columns (360 mg, Milford, MA, USA) separated the samples. The columns were washed with 5 mL of distilled water and 80% MeOH. The eluates were evaporated to dryness, and digoxin was used as the internal standard to determine the purity of fresh and silage AS using ultra-high-performance liquid chromatography with a triple quadrupole mass spectrometer (8890-7000E/PAL RTC120, Agilent Corp., Santa Clara, CA, USA). Extraction yield and post-purification recovery rate were calculated.

#### 2.1.4. Antioxidant Activities

The radical scavenging ability of 1,1-Diphenyl-2-picrylhydrazyl (DPPH) and the ferric reducing ability of plasma (FRAP) were used to determine the antioxidant potential [13]. The samples were redissolved in 16 mL of 80% MeOH. Then, 1 mM antioxidant standard solutions and 200 μL aliquots of the samples were prepared. An amount of 3 mL of a 0.06 mM DPPH solution was added to the samples, and the absorbance at 515 nm was measured every 10 min. Meanwhile, 3 mL of a 0.25 M FRAP solution was added to the samples, and the absorbance at 593 nm was measured every 10 min at 37 °C. Finally, the AS with the lowest and highest antioxidant activity were selected from all 21 alfalfa cultivars.

### 2.2. Enzymatic Biotransformation and Determination

The 2 saponins (Salsa FD4 and Pegasis FD9) mentioned above were used, and their saponins after silage all underwent enzymatic hydrolysis. Salsa (FD4) demonstrated significantly lower antioxidant activity, while Pegasis (FD9) exhibited the highest antioxidant activity. Enzymatic hydrolysis was performed using β-glucosidase (50 U/mg, Shanghai Yuanye Bio-Technology Co., Ltd., Shanghai, China; Cat. No. 230917-2) derived from *A. niger*. Reactions were carried out at 37 °C for 24 h in 50 mM citrate–phosphate buffer (pH 5.0) containing 0.25 U/mL enzyme (0.005 mg/mL) and 1 mg/mL substrate. Control reactions with buffer alone (no enzyme) were included to confirm enzyme-specific activity [14]. After quantitative analyses, the amount of each monosaccharide accounted for one unit of the total glycosides from enzymatically hydrolyzed AS [15]. Following verification of the antioxidant activity and biotransformation, 4 base saponins (FPS: fresh Pegasis saponin; SPS: silage Pegasis saponin; FSS: fresh Salsa saponin; SSS: silage Salsa saponin) and their β-glucosidase-treated derivatives (FPES: fresh Pegasis enzymatic saponin; SPES: silage Pegasis enzymatic saponin; FSES: fresh Salsa enzymatic saponin; SSES: silage Salsa enzymatic saponin) were obtained (see the selection criteria in Section 3.2 and Section 3.3) and used for in vitro rumen fermentation experiments.

Samples were diluted fourfold with ultrapure water containing 2 ng/μL digoxin (internal standard), centrifuged (19,300 r/min, 15 min, 4 °C), and analyzed using two complementary UPLC-QTOF/MS platforms. For the waters’ system analyses, chromatographic separation was achieved on a BEH C18 column (50 °C) with a 0.3 mL/min gradient of 0.1% formic acid in water (A) and acetonitrile (B), programmed from 25% to 100% B over 26 min. Mass spectrometry employed negative ionization (3.1 kV capillary voltage, 140 °C source) with collision energy ramping (5–40 eV) and data-dependent acquisition (*m*/*z* 150–2000). The Dionex–Bruker system utilized an HSS T3 column (40 °C) with 5 mM ammonium formate (A) and acetonitrile (B) at 0.4 mL/min, coupled to an Impact II QTOF operated in ESI ± modes. Solid-phase extraction purification was performed using Oasis HLB cartridges preconditioned with methanol/water, with analytes eluted in 85% methanol, concentrated under nitrogen, and spiked with ^13^C-labeled monosaccharide standards. Final separation employed an XBridge Amide column (35 °C) with 10 mM ammonium formate (pH 4.5)/acetonitrile gradients (85% → 50% B over 15 min) [4].

### 2.3. In Vitro Rumen Fermentation of Goat

The trial was conducted from December 2023 to January 2024 at the goat house, which was cleaned and disinfected with a 3% NaOH solution.

#### 2.3.1. Rumen Fluid Collection and Processing

Six healthy rumen-cannulated female Xuhuai goats (5 years old, body weight 63 ± 2.74 kg) were selected as rumen fluid donors. Goats were housed in individual pens under standardized conditions (temperature: 20–25 °C; humidity: 55–65%; 12 h light/dark cycle) and fed twice daily with a diet containing 80% oat hay and 20% commercial concentrate (soybean meal, wheat bran, corn grain, and mineral vitamins). Feed was provided at 2% DM weight, with ad libitum access to water. All goats were confirmed disease-free prior to the experiment. To ensure biological independence, rumen fluid from each donor goat was processed separately, and fermentations were performed in quartic (*n* = 6 donors × 4 replicates = 24 total runs). Before the morning feeding, ruminal fluid was collected from each goat, filtered through four layers of sterile gauze, and pooled in equal volumes (to minimize individual variability). The pooled fluid was immediately transferred to preheated thermoses saturated with CO_2_ and transported to the laboratory within 30 min.

#### 2.3.2. In Vitro Incubation

The substrate was formulated with 100 mg of oat meal (*Avena sativa* L.) and 100 mg of corn meal (*Zea mays* L.) (DM basis) in several 120 mL anaerobic vials. All vials were sealed with butyl rubber stoppers and aluminum crimp caps to maintain strict anaerobiosis throughout incubation. The ratio of rumen fluid to anaerobic buffer was 1:2, and the buffer contained 1.10 mg/L CaCl_2_⋅2H_2_O, 0.83 mg/L MnCl_2_⋅4H_2_O, 0.08 mg/L CoCl_2_ 6H_2_O, 0.67 mg/L FeCl_3_ 6H_2_O, 5.83 mg/L NaHCO_3_, 0.95 mg/L Na_2_HPO_4_, 1.03 mg/L KH_2_PO_4_, 0.10 mg/L MgSO_4_ 7H_2_O, and 1.25 g/L Na_2_S [16]. The incubation consisted of two parts: (1) the combined AS, which equally represented 21 alfalfa cultivars, was added to the substrates at concentrations of 0, 1.5, 3.0, 6.0, and 9.0 mg; (2) 3.0 mg of FPS, SPS, FSS, SSS, FPES, SPES, FSES, and SSES were added to the substrates. Then, 30 mL of culture media with substrates was incubated in vials under a constant flow of O_2_-free CO_2_ at 39 °C for 48 h (Part 1) and 24 h (Part 2). Vials were manually shaken twice daily (09:00 and 17:00) to simulate rumen motility and ensure substrate-microbe contact, with no mechanical agitation applied. The AS doses (0, 1.5, 3.0, 6.0, 9.0 mg) were selected based on established ranges for saponin efficacy in ruminant in vitro models, where doses ≤50 mg/g DM are commonly reported to suppress methanogenesis without impairing fermentation [17].

#### 2.3.3. Fermentation Parameters

The gas pressure at 0, 3, 6, 9, 12, 24, 36, and 48 h was measured using a digital pressure transducer (DPG1000B15PSIG-5, CeComp Electronics Inc., Chicago, IL, USA). The pH value was measured immediately using a pH meter (PB-21 type, Sartorius, Göttingen, Germany). At 24 and 48 h, the concentrations of CO_2_, H_2_, CH_4_, and N_2_ were analyzed using a GCMS 9800 gas chromatography system (Shanghai Kechuang Chromatographic Instrument Co., Ltd., Shanghai, China) under the following conditions: capillary column (30 cm × 25 mm × 0.25 μm), column temperature of 110 °C (isothermal), injector temperature of 200 °C, thermal conductivity detector temperature of 200 °C, bridge current of 50 mA, and high-purity argon as the carrier gas at a flow rate of 20 mL/min. Quality control (QC) measures for gas analysis included daily calibration with standard gas mixtures (CO_2_, H_2_, CH_4_, N_2_; National Institute of Metrology, Beijing, China) at five concentrations (0.1–10% *v*/*v*), system suitability tests using a certified gas standard (15% CH_4_, 10% CO_2_, 5% H_2_, balanced with N_2_) analyzed every 10 samples, retention time RSD (relative standard deviation) < 1%, peak area RSD < 3%, and blank injections of high-purity argon to confirm absence of column contamination.

For volatile fatty acid (VFA) determination, an 800 μL sub-sample of the removed incubation content was taken at 24 h, mixed with 800 μL of an acid solution (0.5 M HCl, 200 g/L metaphosphoric acid, and 0.8 g/L crotonic acid as an internal standard), and analyzed by GCMS 9800 [17]. The conditions included an injector temperature of 220 °C, a column temperature programmed from 80 °C (hold 1 min) to 125 °C at 3 °C/min, a flame ionization detector at 220 °C, and high-purity nitrogen as the carrier gas at 1.2 mL/min. The QC measures for VFA analysis comprised daily calibration curves with freshly prepared VFA standards (acetate, propionate, butyrate, valerate; Sigma-Aldrich) at five concentrations (0.1–10 mM; R^2^ > 0.995), monitoring of crotonic acid internal standard recovery (85–115% acceptance range), and triplicate analysis of pooled rumen fluid QC samples per batch (inter-day RSD < 8%).

Ammonia-N concentration was determined using the phenol–sodium hypochlorite method [18]. The microbial protein (MCP) was determined using a Spark microplate reader (Tecan, Männedorf, Zürich, Switzerland) [19]. The intracellular Adenosine 5′-triphosphate (ATP) content was measured with a Solarbio detection kit (Beijing, China). DM digestibility was calculated by weighing the dried residue [17].

#### 2.3.4. Quantitative Real-Time Polymerase Chain Reaction (qPCR) of Ruminal Bacteria

Extracellular total DNA in Part 2 was isolated from the fermentation fluid samples using the Solarbio stool genomic DNA extraction kit. Before the procedure, a 3 mL sample was centrifuged at 12,000× *g* for 1 min at 4 °C, and the supernatant was discarded. The primers were synthesized by Qingke Biotech Co., Ltd. (Nanjing, China) (Table 2). The qPCR analyses were performed using the AceQ qPCR SYBR Green Master Mix with the QuantStudio3 instrument (Applied Biosystems, Waltham, MA, USA). The conditions were 95 °C for 5 min, followed by 40 cycles of denaturation at 95 °C for 10 s, and annealing at 60 °C for 30 s. Dissolution curves were obtained by subjecting the samples for 1 cycle of 95 °C for 15 s, followed by 60 °C for 60 s, and concluding with 95 °C for 15 s. To ensure assay specificity and reproducibility, no-template controls were included in every PCR run, and the melt curve analysis confirmed single amplicon peaks for all targets. Each sample was analyzed in triplicate, with inter-run variability minimized by fresh standard curve preparation for every qPCR plate. Finally, the Ct values were calculated using the 2^−ΔΔCT^ method [20].

For each qPCR plate, a new dilution series was prepared from a stock plasmid solution with a concentration of 10^6^ plasmids/μL to generate standard curves. The standard curves were as follows: general bacteria: Y = −3.53 log(X) + 7.08; methanogens: Y = −3.61 log(X) + 9.25; general anaerobic fungi: Y = −3.52 log(X) + 11.42; *F. succinogenes*: Y = −3.73 log(X) + 8.48; *R. albus*: Y = −3.81 log(X) + 10.41; *R. flavefaciens*: Y = −3.69 log(X) + 13.92; *S. bovis*: Y = −3.59 log(X) + 7.08; *B. fibrisolvens*: Y = −3.58 log(X) + 8.27; *S. ruminantium*: Y = −3.93 log(X) + 14.89; *R. amylophilus*: Y = −3.41 log(X) + 7.72; and *P. ruminicola*: Y = −3.74 log(X) + 9.14. Y represents the Ct value, while X represents the log of the concentration of the plasmid. All the R^2^ values were above 0.99, and the amplification efficiency fell within the range of 85% to 115%.

#### 2.3.5. Untargeted Metabolomics Analysis

An amount of 100 μL of fermentation fluid samples were mixed with 400 μL of the extraction solution containing deuterated internal standards (MeOH: ACN, 1:1, *v*/*v*), including L-2-chlorophenylalanine and 4-nitrobenzoic acid for retention time correction and signal normalization), and then centrifuged at 15,000× *g* at 4 °C for 20 min. To ensure reproducibility, a pooled QC sample, generated by combining equal volumes of all experimental samples, was analyzed every 10 injections to monitor instrument stability. Additionally, solvent blanks (extraction solution without sample) were run at the beginning and end of each batch to identify background contamination. The supernatant was diluted to 53% methanol with water, then centrifuged at 15,000× *g* at 4 °C for 20 min. The supernatant was analyzed using a Vanquish ultra-performance liquid chromatography system, coupled to Orbitrap Q Exactive HF-X mass spectrometer (Thermo Fisher Scientific, Niedersachsen, Germany). Data analysis was conducted using the internal standard normalization method. Compound Discoverer 3.3 (Thermo Fisher Scientific) and the mzCloud, mzVault, and MassList (https://www.mzcloud.org) databases were used for raw peaks exaction, data baseline filtering, and calibration of the baseline, peak alignment, deconvolution analysis, peak identification, and integration of the peak area [21]. Metabolites with relative standard deviation (RSD) >30% in the QC samples were excluded to ensure data reliability. Batch effects were corrected using QC-based LOESS normalization. These metabolites were annotated using the Kyoto Encyclopedia of Genes and Genomes (KEGG) database (https://www.genome.jp/kegg/pathway.html, 12 June 2024). The variable N represents the total number of metabolites involved in the KEGG metabolic pathway, *n* denotes the number of differential metabolites among those N, y indicates the number of metabolites annotated to a single KEGG pathway, and x refers to the number of differential metabolites enriched in that KEGG pathway. When the ratio was satisfied by x/*n* > y/N, metabolic pathways were considered as enrichment. The metabolites with VIP (variable importance in projection) >1, *p* < 0.05, and fold change ≥1.50 or ≤0.67 were considered differential metabolites.

### 2.4. Statistical Analysis

Data normality (Shapiro–Wilk test) and variance homogeneity (Levene’s test) were verified, with non-normal parameters log-transformed or analyzed via Duncan’s tests. Dose–response effects were assessed using one-way ANOVA (analysis of variance) with Tukey’s HSD (honestly significant difference) (*p* < 0.05) for pairwise comparisons and orthogonal polynomial contrasts for quadratic trends. Effect sizes (partial eta-squared, η^2^ ≥ 0.06) and residual diagnostics ensured model validity. For multi-omics integration: microbial abundance correlations used Spearman’s rank with the Benjamini–Hochberg FDR (*q* < 0.05); metabolomic pathways were analyzed via PLS-DA (partial least-squares discriminant analysis) (metaX package) with permutation testing (*n* = 1000). Results are reported as a mean ± SD (standard deviation), visualized using Origin 2024.

## 3. Results

### 3.1. Chemical Composition of Fresh and Ensilaged Alfalfa

The chemical composition of fresh alfalfa is presented in Table 3. The DM content across cultivars ranged from 24.09% to 26.38%. The contents of CP and EE decreased with an increase in FD, while ADF showed the opposite trend (*p* < 0.05). While a slight variation was observed in NDF, the difference was generally minor.

For silage alfalfa (Table 4), the CP content increased in most cultivars, except for MS-5S43, Level 6, and Sardi 7II, compared to fresh materials. While the EE, NDF, and ADF were decreased. Overall, the nutrient profiles were relatively stable between fresh and silage alfalfa. The pH values across all silage cultivars ranged from 4.63 to 4.76, and ammonia-N levels were lower than 2.81% for DM. The lactic acid content peaked in Vision (5.90% DM, *p* < 0.05), significantly exceeding Blue Moon, Claudia, Gannong No.5, Eureka+, Pegasis, and Sardi 10. The acetic acid content ranged from 1.21 to 1.55% of DM across cultivars. Butyric acid remained undetectable, confirming anaerobic integrity. These findings indicated standardized ensiling processes.

### 3.2. Antioxidant Activity of Fresh and Silage AS

**Figure 1:**
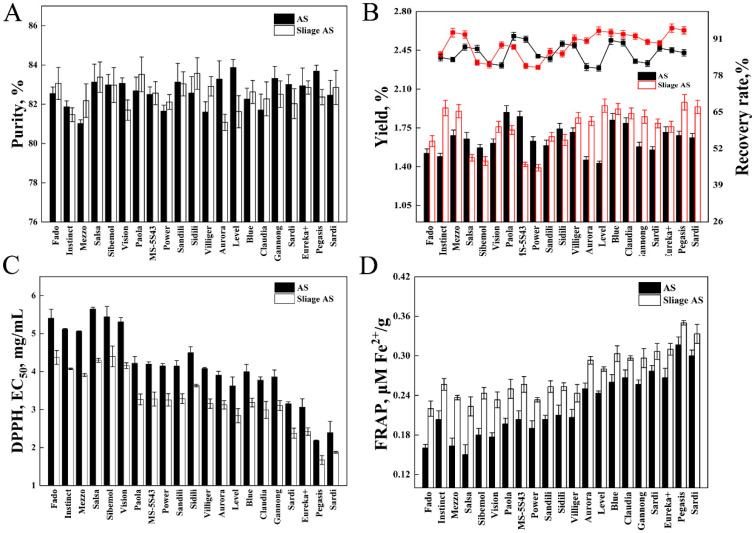
AS (alfalfa saponins) from 21 fresh alfalfa cultivars and their silage. (**A**) Final purity. (**B**) Yield and recovery rate. (**C**) DPPH (1,1-Diphenyl-2-picrylhydrazyl) radical scavenging activities. (**D**) FRAP (ferric reducing ability of plasma) levels. Error bars represent SD (*n* = 4).

The purity assessment was conducted to ensure the extracted AS met quality standards for subsequent animal feeding trials, while minimizing potential interference from co-extracted compounds. The final purity of fresh and silage AS was over 80% (Figure 1A), while the purity of the combined AS reached 82.51%. The AS extraction yields ranged from 1.39% of DM to 1.98% of DM, while the post-purification recovery rates for the AS varied between 74.8% and 89.1% (Figure 1B). The highest half maximum effective concentration (EC_50_) value of DPPH was obtained from FSS (FD4), while the lowest was found in the SPS (FD9) (*p* < 0.05) (Figure 1C). With increasing FD, the EC_50_ value of DPPH showed a decreasing trend. After ensiling, the EC_50_ value of DPPH in FD4 dropped more compared to FD10 (*p* < 0.05). No significant differences were observed in FD9 and FD10 AS for both fresh and silage material.

After ensiling, there was a small increase in the FRAP value (Figure 1D). The SPS (FD9) had the highest FRAP value (*p* < 0.05). In FD5 to FD8, there were no significant differences between fresh and silage AS. Salsa (FD4) and Pegasis (FD9) had FRAP values that increased by 0.07 μM Fe^2+^/g and 0.03 μM Fe^2+^/g. Pegasis (FD9) had the lowest EC_50_ value and the highest FRAP value, indicating the best antioxidant activity, in contrast to Salsa (FD4).

### 3.3. Saponin Glycosylation Conservation via Mass Spectrometry

**Figure 2:**
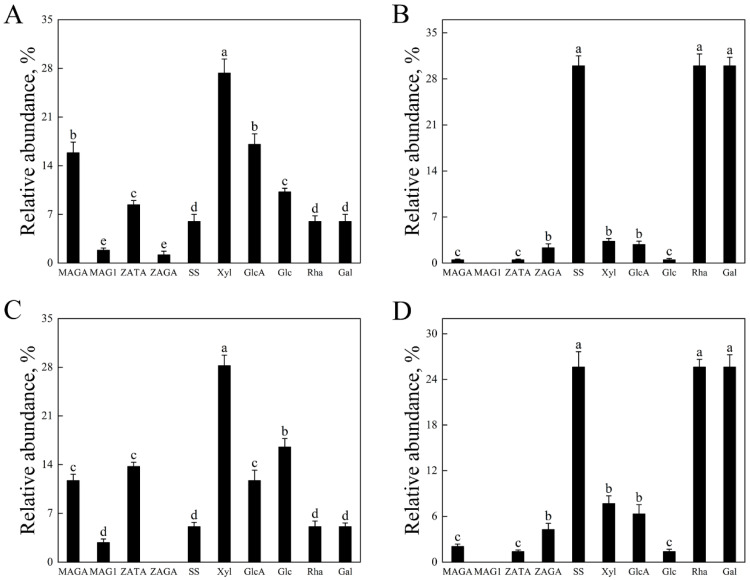
The relative abundance of AS (alfalfa saponins) and their enzymatic hydrolysate-released monosaccharides: (**A**) FPS (fresh Pegasis saponin) and FPES (fresh Pegasis enzymatic saponin); (**B**) SPS (silage Pegasis saponin) and SPES (silage Pegasis enzymatic saponin); (**C**) FSS (fresh Salsa saponin) and FSES (fresh Salsa enzymatic saponin); (**D**) SSS (silage Salsa saponin) and SSES (silage Salsa enzymatic saponin). Different lowercase letters indicate statistically significant differences (*p* < 0.05, one-way ANOVA; Tukey’s HSD). Error bars represent SD (*n* = 4). Enzyme-to-substrate ratio: 0.25 U β-glucosidase per mg saponin (1 mg/mL substrate in 50 mM citrate-phosphate buffer). Abbreviations: MAGA (1087.49 Da), 3-GlcA-28-AraRhaXyl medicagenic acid; MAG1 (1073.54 Da), 3-Glc-28-AraRhaXyl medicagenic acid; ZATA (1087.49 Da), 3-Glc-28-AraRhaAraXyl Zanhic acid; ZAGA (1103.49 Da), 3-GlcA-28-AraRhaXyl Zanhic acid; SS (942.52 Da), 3-GlcAGalRha soyasapogenol B; Xyl (150.13 Da), Xylose; GlcA (194.14 Da), Glucuronic acid; Glc (180.16 Da), Glucose; Rha (164.15 Da), Rhamnose; Gal (180.16 Da), Galactose.

Mass spectrometric analysis demonstrated that β-glucosidase hydrolysis of fresh and silage-derived saponins generated deglycosylated derivatives respectively. Comparative quantification revealed structural conservation of specific sugar moieties post-hydrolysis. In the FPES (Figure 2A), xylose content matched the combined xylose groups of MAGA, MAG1, ZATA, and ZAGA in the FPS, while its glucuronic acid corresponded to the sum of MAGA and ZAGA in the FPS. Glucose in the FPES aligned exclusively with MAG1 and ZATA in the FPS, and rhamnose/galactose levels mirrored those of SS in the FPS. In silage-derived analogs, the SPES exhibited xylose levels matching MAGA/ZATA/ZAGA in the SPS, glucuronic acid mirroring MAGA/ZAGA, glucose content similar to ZATA, and rhamnose/galactose aligned with SS (Figure 2B). The FSES exhibited xylose content equivalent to MAGA, MAG1, and ZATA in the FSS, with glucuronic acid derived solely from MAGA (Figure 2C). Its glucose content paralleled MAG1 and ZATA in the FSS, and rhamnose/galactose matched SS in the FSS. The SSES displayed analogous patterns, with xylose and glucuronic acid corresponding to MAGA, ZATA, and ZAGA in the SSS, glucose content paralleled ZATA, while rhamnose/galactose aligned with SS in the SSS (Figure 2D). These findings confirm the systematic retention of glycosyl group stoichiometry during enzymatic processing across all saponin variants. Enzymatic hydrolysis achieved 82–89% conversion yield (calculated as hydrolyzed saponin mass/initial saponin mass) with ≥95% purity for all derivatives.

### 3.4. Fermentation Parameters of the Optimum Addition of AS

**Figure 3:**
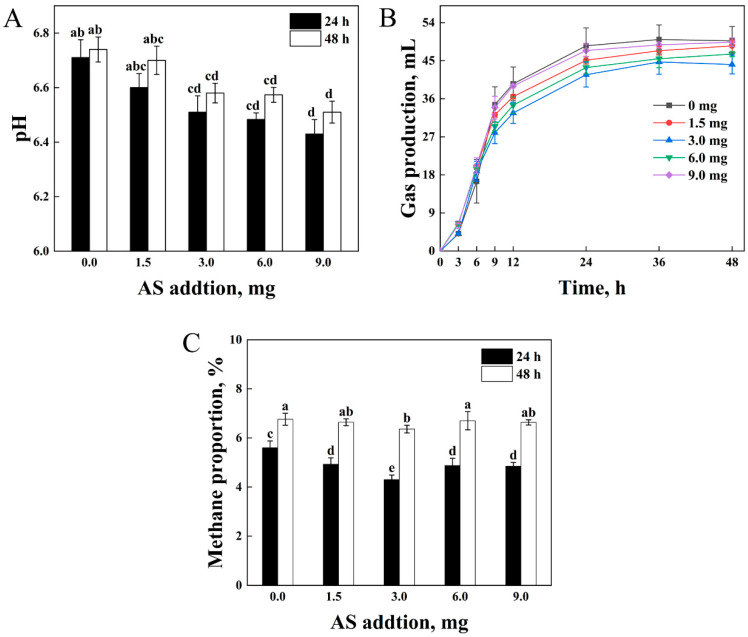
Fermentation parameters of the combined AS (alfalfa saponins) in vitro at levels of 0 (Control), 1.5, 3.0, 6.0, and 9.0 mg. (**A**) pH value. (**B**) Gas production. (**C**) Methane proportion. Different lowercase letters indicate statistically significant differences (*p* < 0.05, one-way ANOVA; Tukey’s HSD). Data are presented as a mean ± SD (*n* = 4).

During the in vitro incubations, β-glucosidase from *A. niger* did not remain active. As the dose of AS increased, the pH value decreased (Figure 3A). Gas production exhibited an increasing trend from 0 to 24 h and then plateaued until 48 h (Figure 3B). From 24 to 48 h, the lowest gas production was detected at 3.0 mg for AS, while the highest was 6.0 mg (*p* < 0.05). The methane proportion decreased at all AS levels compared to the blank (*p* < 0.05) (Figure 3C). A non-linear dose–response relationship was observed, with maximal methane reduction (30.15%) at 3.0 mg for AS. Polynomial contrast analysis revealed a significant quadratic trend (*p* < 0.05, η^2^ = 0.20). Higher doses (6.0, 9.0 mg) showed diminished effects (14.86%, 15.44%), suggesting a potential threshold for saponin efficacy. The moderate effect size (η^2^ = 0.20) indicates biological relevance despite marginal statistical significance.

### 3.5. Fermentation Parameters of the AS

**Figure 4:**
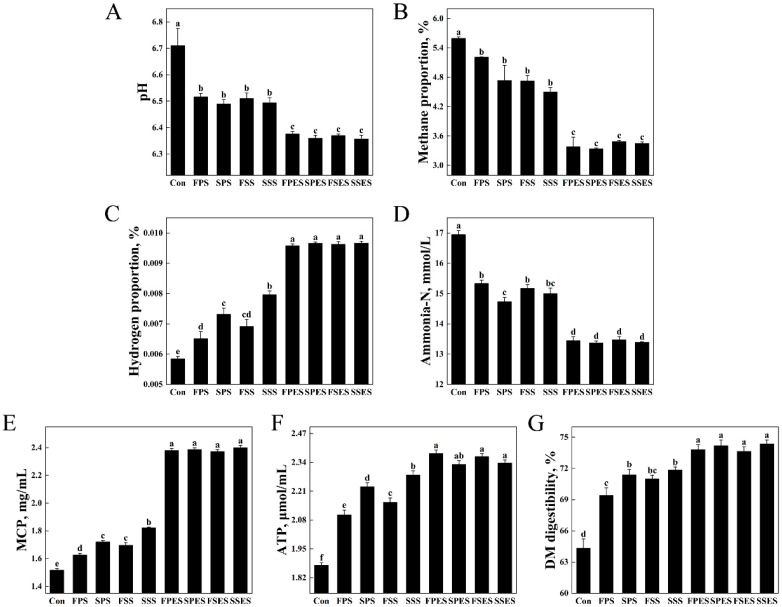
Fermentation parameters of the AS (alfalfa saponins) in vitro after 24 h. (**A**) pH value. (**B**) Methane proportion. (**C**) Hydrogen proportion. (**D**) Ammonia-N concentration. (**E**) MCP (microbial protein) concentration. (**F**) ATP (adenosine 5′-triphosphate) concentration. (**G**) DM (dry matter) digestibility. Control (Con) was blank treatment, while experimental groups (FPS, SPS, FSS, SSS, FPES, SPES, FSES, SSES) contained 3 mg of AS and 200 mg of fermentation substrate. Different lowercase letters indicate statistically significant differences (*p* < 0.05, one-way ANOVA; Tukey’s HSD). Data are presented as a mean ± SD (*n* = 4). Abbreviations: FPS, fresh Pegasis saponin; SPS, silage Pegasis saponin; FSS, fresh Salsa saponin; SSS, silage Salsa saponin; FPES, fresh Pegasis enzymatic saponin; SPES, silage Pegasis enzymatic saponin; FSES, fresh Salsa enzymatic saponin; SSES, silage Salsa enzymatic saponin.

The pH value ranged between 6.30 and 6.60 (Figure 4A). After enzymatic biotransformation, the methane proportion significantly decreased compared to that of fresh and silage AS (*p* < 0.05) (Figure 4B). No significant differences were detected among the FPES, SPES, FSES, and SSES groups. Enzymatic AS supplementation significantly increased the hydrogen proportion (*p* < 0.05), with the FPS showing lower values than the SPS (*p* < 0.05) (Figure 4C). Ammonia-N concentration was significantly reduced by enzymatic AS (*p* < 0.05), where the FPS and the FSS exceeded the SPS (*p* < 0.05) (Figure 4D). The MCP yields in the FPES, SPES, FSES, and SSES groups (2.08–2.10 mg/mL) represented significant increases of 31.81–46.26% compared to controls (*p* < 0.05) (Figure 4E). ATP concentration was significantly elevated by the FPES, FSES, and SSES, while the SSS surpassed the FPS, SPS, and FSS (*p* < 0.05) (Figure 4F). DM digestibility was markedly improved by AS addition, particularly in enzymatic treatment groups (*p* < 0.05) (Figure 4G).

The addition of AS resulted in a highly significant increase in total VFA production (*p* < 0.05) (Table 5). The total VFA, molar percentage of acetate and iso-butyrate, and acetate to propionate ratio in the FPES, SPES, FSES, and SSES were statistically distinct from those in the FPS, SPS, FSS, and SSS (*p* < 0.05). Fresh AS showed 1.53–2.84 mM lower total VFA than silage AS (*p* < 0.05), but higher molar percentages of acetate (1.70–2.14%) and iso-butyrate (1.91–4.64%), along with a 5.42–6.33% greater acetate to propionate ratio (*p* < 0.05). The molar percentages of propionate and butyrate showed significant but less pronounced increases after the addition of the FPES, SPES, FSES, and SSES (*p* < 0.05).

### 3.6. Microorganisms of the AS

**Figure 5:**
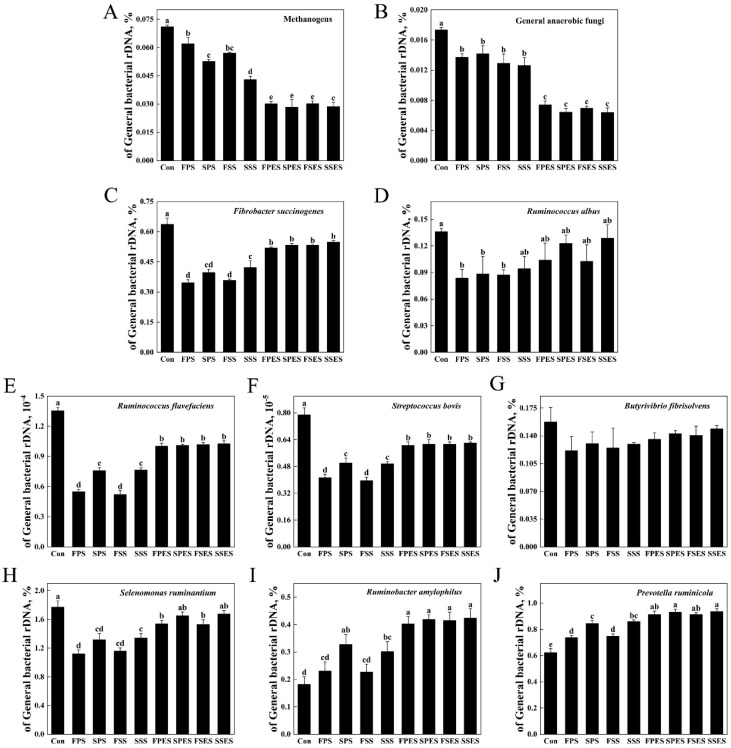
Changes in target microorganisms of the AS in vitro as a proportion of total general bacteria. (**A**) Methanogens. (**B**) General anaerobic fungi. (**C**) *Fibrobacter succinogenes*. (**D**) *Ruminococcus albus*. (**E**) *Ruminococcus flavefaciens*. (**F**) *Streptococcus bovis*. (**G**) *Butyrivibrio fibrisolvens*. (**H**) *Selenomonas ruminantium*. (**I**) *Ruminobacter amylophilus*. (**J**) *Prevotella ruminicola*. Control (Con) was blank treatment, while experimental groups (FPS, SPS, FSS, SSS, FPES, SPES, FSES, SSES) contained 3 mg of AS and 200 mg of fermentation substrate. Different lowercase letters indicate statistically significant differences (*p* < 0.05, one-way ANOVA; Tukey’s HSD). Data are presented as a mean ± SD (*n* = 4). Abbreviations: FPS, fresh Pegasis saponin; SPS, silage Pegasis saponin; FSS, fresh Salsa saponin; SSS, silage Salsa saponin; FPES, fresh Pegasis enzymatic saponin; SPES, silage Pegasis enzymatic saponin; FSES, fresh Salsa enzymatic saponin; SSES, silage Salsa enzymatic saponin.

A microbial community analysis revealed a significant decrease in the relative abundances of methanogens and general anaerobic fungi in the FPES, SPES, FSES, and SSES compared to the FPS, SPS, FSS, and SSS (*p* < 0.05) (Figure 5). A statistical analysis showed a significant enhancement in the relative abundances of *F. succinogenes*, *R. flavefaciens*, *S. bovis*, and *P. ruminicola* in the SSS compared to the FPS and FSS (*p* < 0.05). The relative abundances of *S. ruminantium* in the SSS was significantly higher than in the FPS (*p* < 0.05), while *R. amylophilus* in the SPS showed significantly higher abundance compared to the FPS and FSS (*p* < 0.05).

### 3.7. Correlation Analysis of Fermentation Parameters and Microorganisms

**Figure 6:**
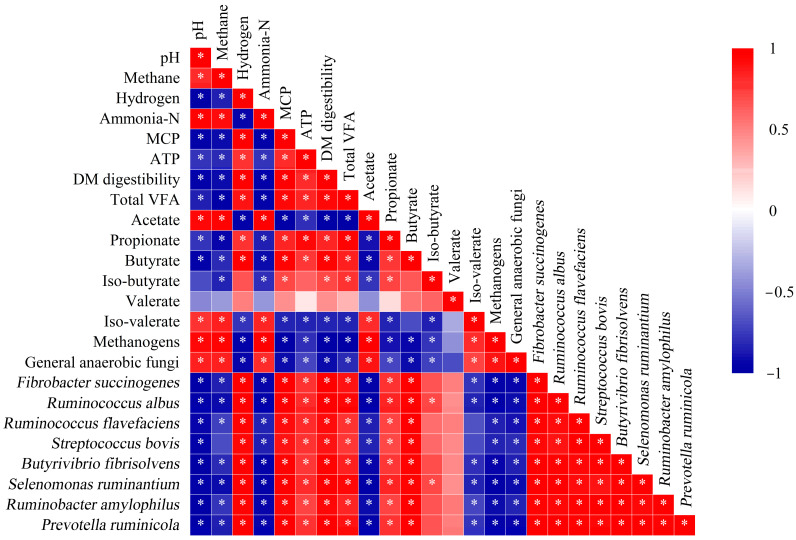
Spearman correlation heatmap of the relative abundances of fermentation parameters and microbial populations. * *p* < 0.05. Abbreviations: MCP, microbial protein; ATP, adenosine 5′-triphosphate; DM, dry matter; VFA, volatile fatty acid.

Methane showed positive correlations with acetate and methanogens (Spearman’s ρ > 0.7, *p* < 0.05), while exhibiting negative correlations with total VFA and propionate (ρ < −0.81, *p* < 0.05) (Figure 6). A negative correlation was observed between microbial abundance (methanogens, general anaerobic fungi) and the MCP yield (−0.65 < ρ < −0.52, *p* < 0.05). Meanwhile, *F. succinogenes*, *R. albus*, *R. flavefaciens*, *S. bovis*, *B. fibrisolvens*, *S. ruminantium*, and *R. amylophilus* showed a positive correlation with the MCP yield (0.42 < ρ < 0.58, *p* < 0.05). Ammonia-N demonstrated significant positive correlations with pH, acetate, and methanogens (ρ = 0.65–0.82, *p* < 0.05). The DM digestibility showed significant negative correlation with methane (ρ = −0.42) and methanogens (ρ = −0.78), and positive correlation with total VFA (ρ = 0.81) (*p* < 0.05). Notably, the pH was negatively associated with multiple microbial species, including *F. succinogenes*, *R. albus*, *R. flavefaciens*, *S. bovis*, *B. fibrisolvens*, *S. ruminantium*, *R. amylophilus*, and *P. ruminicola* (all ρ < −0.7, *p* < 0.05).

### 3.8. Rumen Fluid Metabolome

**Figure 7:**
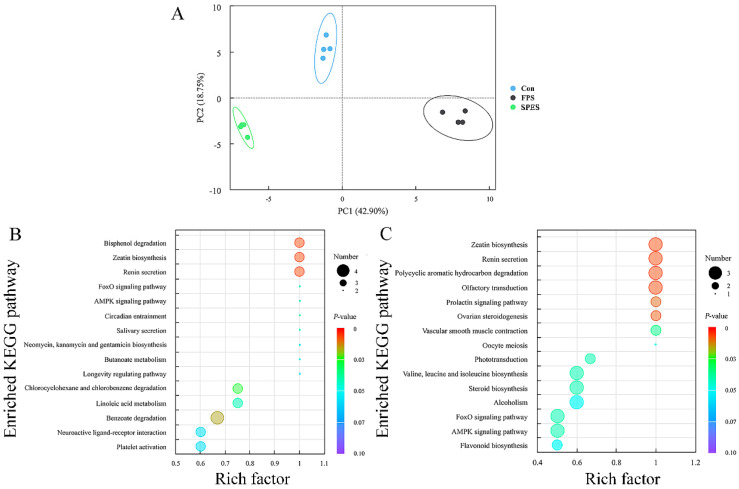
The metabolites of rumen fluid. (**A**) PLS-DA (partial least-squares discriminant analysis) models. KEGG (Kyoto Encyclopedia of Genes and Genomes) pathways of the (**B**) FPS (fresh Pegasis saponin) and (**C**) SPES (silage Pegasis enzymatic saponin) visualized in bubble plots (*p* < 0.05). Multivariate model validation confirmed robustness with cross-validated Q^2^ values > 0.75 for all significant parameters (*n* = 4).

**Figure 8:**
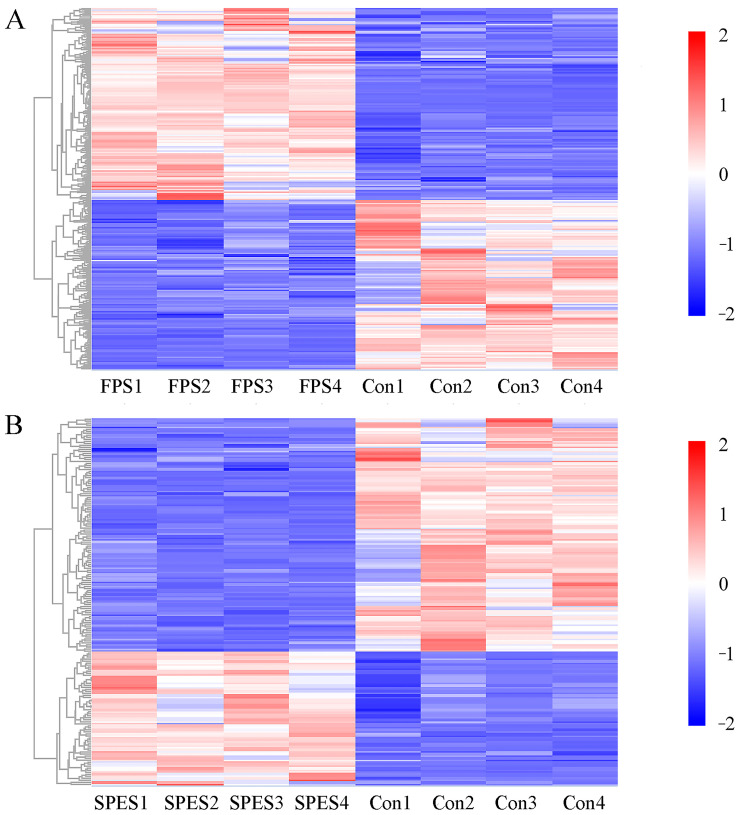
Hierarchical clustering heatmap of metabolite abundance in (**A**) FPS and (**B**) SPES treatments (*n* = 4).

The methane proportion was lowest in the SPES and highest in the FPS (*p* < 0.05), suggesting that saponin treatments differentially regulate methanogenesis. An untargeted metabolomics analysis revealed distinct metabolite profiles across the treatments, with PLS-DA confirming significant differences (*p* < 0.05) and clear cluster separation (Figure 7A). In the FPS, the most altered pathways included bisphenol degradation and zeatin biosynthesis, characterized by increased antimicrobial phenolic compounds (4-hydroxybenzoic acid and hydroquinone) and decreased nucleotide derivatives (adenosine monophosphate, adenosine diphosphate, and guanosine-3′,5′-cyclic monophosphate; Figure 8).

The SPES exhibited metabolic remodeling in polycyclic aromatic hydrocarbon degradation and ovarian steroidogenesis, with significant upregulation of steroidal metabolites (e.g., estrone, progesterone, dehydroepiandrosterone; VIP > 1.0, FDR < 0.05). These steroids may directly inhibit methanogens by destabilizing membrane lipids or indirectly suppress interspecies hydrogen transfer via reduced ATP and guanosine derivatives. Notably, both saponin treatments reduced bisphenol A, a xenobiotic linked to methanogen proliferation, further supporting their anti-methanogenic potential (Figure 7B,C).

Dendrograms reflect similarity in metabolite profiles, with clusters highlighting metabolites co-regulated under saponin treatments. Key anti-methanogenic metabolites (e.g., phenolic acids, steroidal compounds) are enriched in distinct branches, aligning with their roles in bisphenol degradation and steroidogenesis pathways. Red/blue gradients denote up-/downregulation relative to the control.

## 4. Discussion

Plant secondary metabolites have been widely studied for their potential therapeutic and health benefits, including antioxidant, antimicrobial, and anti-inflammatory properties, due to their availability, safety, and non-toxicity [22]. Saponins from alfalfa extracts consistently affected the in vitro rumen fermentation, as well as methane and metabolite changes [2], particularly after hydrolysis by β-glucosidases.

### 4.1. The Selection of AS

Despite alfalfa’s inherent ensiling challenges (low WSC, high buffering), controlled fermentation succeeded, evidenced by optimal pH (4.63–4.76) and lactic acid dominance (5.90% DM in Vision), confirming effective homolactic activity [23]. The absence of butyric acid validated anaerobic integrity. Notably, ammonia-N levels (<2.81% DM) fell below typical proteolysis ranges (5–7% total N), potentially due to the enhanced fermentation efficiency of lactic acid bacteria additives, which suppress proteolysis and stabilize nutrients. The CP declined with FD (FD4:23.53% vs. FD10:18.06%), aligning with maturity-driven lignification [3]. The EE reduction and ADF elevation indicated cell wall degradation, and acetic acid (1.21–1.55% DM) supported aerobic stability with less quality loss [24].

The antioxidant activities of saponins extracted from plants are usually determined by DPPH and FRAP tests, and critically influence rumen redox homeostasis by protecting fiber-degrading microbes from oxidative stress [5]. In the DPPH test, the ability of the extracts to neutralize free radicals was estimated. In the FRAP test, antioxidant compounds donate a single electron to the FRAP reagent. Both saponins from *Crocus sativus* L. [25] and *Ganoderma applanatum* [26] are primarily triterpenoid, similar to AS, and include a hydrophilic part (several saccharide residues) linked to a hydrophobic scaffold through glycosidic bonds. Salsa (FD4) exhibited the lowest antioxidant activities, whereas Pegasis (FD9) demonstrated the highest antioxidant activities. The antioxidant activities of the AS are attributed to phenolic compounds, and due to interactions with these compounds, the AS remain particularly effective after purification [27]. Saponin exhibits thermal stability and bioactivity, with an increase in antioxidant activities observed after ensiling. During ensiling, enzymatic activities may release bindings and increase saponin availability. The antioxidant activities among different cultivars may be associated with the FD of alfalfa [28]. Additionally, the harvesting time and weather were important factors; thus, the antioxidant activity of the AS from Sardi 10 (FD10) was not the highest.

Adding AS significantly reduced methane emissions. While the observed methane percentage (5.60% vs. in vivo 26–28%) reflects methodological constraints of the 24 h incubation system, two potential mechanisms are proposed. First, the initial CO_2_; flushing may dilute subsequent methane accumulation, and residual CO_2_ could theoretically serve as a substrate for methanogens. However, short-term anaerobic fermentation likely limited methanogen activity under these conditions [29]. Second, the methane-suppressive effects of AS primarily stem from their disruption of protozoa–methanogen symbiosis, as evidenced by reduced methanogen abundance and suppressed hydrogenotrophic methanogenesis pathways [30]. By adding 1 mg/mL *Quillaja* saponins to a 100 mg/g DM substrate, Castro-Montoya et al. [31] noted a 25% decrease in methane proportion, and the doses exhibited inhibitory effects. After 6 h of incubation, the addition of 3.0 mg of AS showed better inhibition gas production, suggesting that the initial methane-suppressing effect of AS was transient. After adding AS, the pH ranged from 6.43 to 6.70, indicating that the ruminal environment was maintained in a stable condition [32]. The decrease in pH and the increase in total gas production with increasing AS addition compared to the control are due to AS altering the microbial composition and increasing membrane permeability, which leads to microbial activities that produce organic acids as metabolic byproducts, suggesting accelerated substrate utilization [2]. Meanwhile, the 3.0 mg of AS consistently demonstrated the lowest gas production from 9 to 48 h, and it showed the lowest methane proportion at 24 h. The transient methane increase at higher AS doses (6.0–9.0 mg) may arise from microbial detoxification processes that temporarily release methanogenic substrates (e.g., methyl groups) [17], or redirected hydrogen flux toward residual methanogen populations when partial inhibition occurs [33]. The stronger suppression at 3.0 mg of AS likely represents an optimal dose where saponin efficacy outweighs microbial adaptive responses, consistent with the phytochemicals’ biphasic effects on methane mitigation [34]. Thus, the 30.15% methane reduction achieved by 3.0 mg of enzymatic AS suggests its potential as a cost-effective feed additive for sustainable goat production systems.

### 4.2. Enzymatic AS Modulates Rumen Fermentation

The minimal pH shifts post-AS addition suggest negligible disruption to ruminal fermentation. Critically, β-glucosidase from *A. niger* hydrolyzes β-glycosidic bonds in AS triterpenoid glycosides [8], cleaving sugar moieties to release structurally similar aglycones (pentacyclic triterpenoid cores) [15]. This enzymatic modification explains the consistent methane suppression patterns across AS treatments: the shared aglycone structure likely drives uniform interactions with methanogen membranes or hydrogenase enzymes, independent of original glycosylation differences. Hydrogen produced during VFA fermentation serves as a key substrate for methanogens, directly linking VFA profiles to methane yield. The significant variations in butyrate across treatments are particularly relevant: butyrate synthesis via the butyryl-CoA: acetate CoA-transferase pathway generates excess hydrogen, which typically promotes methanogenesis [33]. Enzymatic AS enhanced the proportion of hydrogen while reducing the proportion of methane and the conversion of hydrogen to methane, which led to the inhibition of methanogens. Batch incubation in vitro eliminated the influence of physiological conditions [35].

In ruminant digestion, lignocellulose is degraded into soluble sugars and then converted into VFA for energy metabolism. The substrate is formulated with oat and corn meal, containing nitrogen-free extract and starch, which can be rapidly and completely fermented into VFA. In the fermentation of glucose to acetate, hydrogen is usually produced, while in the fermentation of glucose to propionate, hydrogen is incorporated [36]. The enzymatic AS increased the molar percentage of propionate while decreasing the molar percentage of acetate and the acetate to propionate ratio. Thus, enzymatic AS altered the fermentation pathway from acetate production to propionate production. Hydrogen accumulation directly correlated with methane suppression and modulated total VFA levels [29].

In the process of rumen fermentation in goats, the production of methane and ammonia is energetically wasteful and harmful. The ammonia-N concentration decreased with enzymatic AS supplementation, suggesting an attenuation of microbial proteolytic activity [37] and an ammonia-binding property of saponins [19]. The ammonia-N concentration decreased, and the MCP yield increased with the addition of the FPES, SPES, FSES, and SSES. Mao et al. [38] showed that ciliate protozoa contribute to the cycling of microbial nitrogen and the synthesis of MCP in the rumen. Thus, the addition of enzymatic AS can improve the MCP flow to the intestine. Hydrogen-derived reducing equivalents [H] fueled the ATP synthesis, driving the MCP production [39]. Hydrolysis of AS by β-glucosidases reduced ammonia-N concentration and increased the MCP and ATP yields, thereby altering rumen fluid fermentation.

### 4.3. Rumen Microorganisms and Spearman Correlation

MCP and ammonia-N were metabolized by rumen microbes, and some ammonia-N can be synthesized into MCP. The reduction of ammonia-N production by saponin may not be caused by direct inhibition of proteolytic bacteria. *Streptococcus bovis*, *B. fibrisolvens*, *S. ruminantium*, *R. amylophilus*, and *P. ruminicola* are major known protein-degrading rumen bacteria. The relative abundances of these microorganisms were significantly negatively correlated with ammonia-N production, while the MCP production showed the opposite trend. The most common methanogens found in the rumen belong to the family *Methanobacteriaceae*, and their abundances were inhibited by saponin alone [40]. The silage AS had a stronger inhibitory effect compared to fresh AS. Methane mitigation through the modulation of methanogens in ruminal fluid fermentation may negatively affect cellulolytic bacteria, potentially reducing feed digestibility [41]. Additionally, the relative abundance of methanogens was significantly negatively correlated with microbial communities involved in feed utilization (eight variables). Higher feed digestibility was associated with increased levels of VFA and reduced methane production.

The methane was extremely significantly positively correlated with acetate and methanogens. Acetate was considered to be an important precursor for methane production, while the methanogens were strict anaerobes with the metabolic capacity to produce methane [42]. The relative abundance of general anaerobic fungi was significantly positively correlated with methane proportion and has shown the ability to degrade various substrates, including AS. The production of methane was demonstrably increased by integrating fungi through bioaugmentation during mainstream processing [43]. Saponin supplementation increased *F. succinogenes* and *R. flavefaciens* abundances, which strongly correlated with cellulolytic partners (*S. bovis*, *S. ruminantium*). This synergy likely enhanced fiber degradation, redirecting hydrogen toward propionate synthesis over methanogenesis. These effects can be attributed to the direct stimulation of cellulolytic bacteria or to defaunation by AS, especially in the β-glucosidase-treated samples. It is important to note that the saponin reduced the proportion of methane, in addition to improving the degradability and fermentation profile of the feed [40]. These results were corroborated by a decrease in the relative abundance of methanogens and methane production, due to the changes in the chemical structure of AS after ensiling or enzymatic hydrolysis. Saponins in low doses increase the permeability of the cell membrane, leading to increased absorption of nutrients into the bacterial cells [44]. The growth of the relative abundances of *S. bovis*, *B. fibrisolvens*, butyrate, and iso-butyrate in enzymatic AS confirmed this. Normally, the growth of *S. bovis* with concentrate-based diets increased [45], and *B. fibrisolvens* played a role in biohydrogenation. *Selenomonas ruminantium* was primarily responsible for the production of propionate from succinate metabolism [46]. The relative abundance of *S. ruminantium* was significantly positively correlated with propionate production and significantly negatively correlated with methane proportion. The formation of ruminal propionate occurs through succinate, acrylate, and propanediol pathways. Due to the energic effect of propionate on goats, redirecting [H] from methane to propionate would be a promising strategy for methane mitigation [47]. The enzymatic stabilization approach could be implemented through mobile processing units or regional hubs, with initial cost estimates suggesting feasibility for medium-scale operations. Further optimization of delivery methods and cost–benefit analyses relative to existing strategies are needed to establish economically viable inclusion levels in goat production systems.

### 4.4. Rumen Metabolomics Are Influenced by FPS and SPES

Metabolomics provides a real-time monitoring approach and plays an integral role in systems biology. Depending on the chromatographic and mass spectrometric techniques applied, 50 to 5000 different metabolites with relative molecular weights of less than 1500 Da can be identified simultaneously in biological samples [48]. The contrasting regulation of zeatin biosynthesis and renin secretion pathways between FPS (downregulated) and FPES (upregulated) may reflect their roles in modulating rumen microbial ecology under stress conditions. Zeatin biosynthesis can modulate bacterial quorum sensing and carbohydrate utilization, while renin secretion pathways may alter sodium flux and rumen osmotic pressure, indirectly shaping microbial niches [49]. The addition of AS changed the yields of adenosine monophosphate, uridine 5′-diphosphate, adenosine diphosphate, and cyclic guanosine monophosphate. In energy metabolism, ruminal bacteria and environmental conditions in the rumen affect the yield of adenosine monophosphate and adenosine diphosphate [39], which in turn affects the yield of ATP. Uridine 5′-diphosphate is associated with glycogen metabolism, and the production of ATP, along with the degradation of glycogen in rumen fluid, influences the rate of pH decrease. Cyclic guanosine monophosphate played a key role in the physiological process of electrolyte transport [50]. The metabolic pathway of bisphenol degradation in the FPS was correlated with the upregulation of 4-hydroxybenzoic acid and hydroquinone. Saponins had a direct effect on methanogens and protozoa, and 4-hydroxybenzoic acid can be converted into phenol and benzoic acid [51]; therefore, further research is needed to describe the phenolic metabolism.

The addition of the SPES showed modulation of pathways including prolactin signaling and ovarian steroidogenesis (consistent with saponin effects observed in murine models [52]). However, direct extrapolation to ruminant physiology requires caution, as these pathways primarily regulate mammalian endocrine functions (e.g., lactation and reproduction). Speculatively, such modulation might influence microbial nutrient utilization through host–microbe crosstalk, though mechanistic validation in ruminants remains needed. The observed upregulation of olfactory transduction (a sensory signaling pathway) could reflect saponin-induced chemosensory responses in rumen epithelia or microbes. While this pathway’s role in rumen fermentation is unclear, improved feed palatability through olfactory mechanisms has been hypothesized but not yet demonstrated in goats. Further studies should assess whether these metabolic shifts correlate with actual feed intake or sensory behavior changes. In total, the SPES has influenced more metabolic pathways in rumen fluid and is more effective than the FPS.

While these metabolomic shifts indicate that enzymatic AS may remodel rumen microbial metabolism, their functional relevance to methane mitigation requires confirmation through integrated host–microbe studies. However, translating these in vitro findings to practical applications requires caution. The absence of host physiological feedback (e.g., endocrine regulation of rumen pH, immune responses to microbial shifts) in the batch culture system may overestimate the efficacy of enzymatic AS [14]. Prospective in vivo trials should monitor whether the observed metabolic pathway modulation (e.g., prolactin signaling inhibition and ovarian steroidogenesis) aligns with changes in animal endocrine profiles or oxidative stress markers. Furthermore, field studies under diverse feeding systems (e.g., high-grain vs. pasture-based diets) are needed to evaluate the robustness of enzymatic AS across real-world scenarios.

### 4.5. Limitations of in Vitro Models and Translational Considerations

The in vitro fermentation system employed in this study provides valuable insights into the methane-suppressing effects of enzymatic AS. However, inherent limitations of batch culture models must be acknowledged. For instance, such systems simplify dynamic interactions between rumen microbiota, host physiology (e.g., immune regulation, hormonal feedback), and long-term adaptive responses. The static nature of the model, which lacks continuous nutrient intake and rumen outflow, may overestimate the persistence of enzymatic AS effects observed in short-term incubations [53]. Additionally, host–microbe cross-talk, which modulates methane production in vivo, is not fully captured in in vitro systems [54]. These factors could contribute to discrepancies between in vitro findings and practical outcomes in ruminants.

To bridge this gap, future studies should prioritize the in vivo trials to validate dose-dependent efficacy, assess long-term safety (e.g., effects on liver function, rumen epithelium health), and evaluate trade-offs between methane mitigation and animal productivity (e.g., weight gain, milk yield). Pilot in vivo experiments could clarify whether metabolic pathway shifts (e.g., prolactin signaling inhibition) observed in this study translate to measurable changes in endocrine profiles or oxidative stress markers. Such translational validation is critical to ensure the practical applicability of enzymatic AS in sustainable ruminant production systems [55]. A concurrent positive control (e.g., monensin) was not included in this study. However, system functionality was indirectly validated through substrate-free controls and dose–response trends. Future studies will incorporate pharmacological controls to further strengthen mechanistic interpretations.

## 5. Conclusions

Silage processing significantly boosted the antioxidant capacity of AS, with Pegasis (FD9) exhibiting the highest activity (EC_50_ 2.13 mg/mL) and Salsa (FD4) the lowest (*p* < 0.05). A 3.0 mg dose of AS reduced methane production to 4.50–5.21% in vitro, which is linked to methanogen inhibition, propionate enrichment via *S. ruminantium* proliferation, and hydrogen flux redirection from methanogenesis to VFA synthesis. *Aspergillus niger* β-glucosidase hydrolysis enhanced AS efficacy, lowering methane to 3.34–3.48% of total gas while maintaining stable pH (6.30–6.45) and DM digestibility. Divergent pathway regulation between the FPS and SPES, notably in zeatin biosynthesis (nucleotide metabolite accumulation) and polycyclic aromatic hydrocarbon degradation (aromatic compound enrichment), highlights the saponin–microbe crosstalk. This study establishes enzymatic alfalfa saponins as a dual-action feed additive for sustainable ruminant production, concurrently mitigating methane emissions and enhancing nutrient utilization. Future work must validate these effects in vivo and optimize delivery systems (e.g., rumen-protected pellets) to translate laboratory efficacy to farm-scale impact.

## Figures and Tables

**Table 1:** Source of tested alfalfa materials (*n* = 4).

No.	Cultivars	Fall Dormancy	Dormancy Category	Origin
1	Fado	4	Semi-dormant	French
2	Instinct	4	Semi-dormant	French
3	Mezzo	4	Semi-dormant	French
4	Salsa	4	Semi-dormant	French
5	Sibemol	4	Semi-dormant	French
6	Vision	4	Semi-dormant	French
7	Paola	5	Moderate	Italian
8	MS-5S43	5	Moderate	American
9	Power 5020	5	Moderate	Canadian
10	Sandili	5	Moderate	American
11	Sidili	5	Moderate	American
12	Villiger	5	Moderate	Canadian
13	Aurora	6	Moderate	Australian
14	Level 6	6	Moderate	American
15	Blue moon	7	Moderate	Italian
16	Claudia	7	Moderate	Italian
17	Gannong No.5	7	Moderate	Chinese
18	Sardi 7	7	Moderate	Australian
19	Eureka+	8	Dormant	Australian
20	Pegasis	9	Dormant	Australian
21	Sardi 10	10	Non-dormant	Australian

**Table 2:** qPCR primers for detection of rumen bacteria.

Primer Name	Sequences
General bacteria-F	CGGCAACGAGCGCAACCC
General bacteria-R	CCATTGTAGCACGTGTGTAGCC
Methanogens-F	CCGGAGATGGAACCTGAGAC
Methanogens-R	CGGTCTTGCCCAGCTCTTATTC
General anaerobic fungi-F	GAGGAAGTAAAAGTCGTAACAAGGTTTC
General anaerobic fungi-R	CAAATTCACAAAGGGTAGGATGATT
*Fibrobacter succinogenes*-F	GCGGGTAGCAAACAGGATTAGA
*Fibrobacter succinogenes*-R	CCCCCGGACACCCAGTAT
*Ruminococcus albus*-F	CCCTAAAAGCAGTCTTAGTTCG
*Ruminococcus albus*-R	CCTCCTTGCGGTTAGAACA
*Ruminococcus flavefaciens*-F	CGAACGGAGATAATTTGAGTTTACTTAGG
*Ruminococcus flavefaciens*-R	CGGTCTCTGTATGTTATGAGGTATTACC
*Streptococcus bovis*-F	TTCCTAGAGATAGGAAGTTTCTTCGG
*Streptococcus bovis*-R	ATGATGGCAACTAACAATAGGGGT
*Butyrivibrio fibrisolvens*-F	GCCTCAGCGTCAGTAATCG
*Butyrivibrio fibrisolvens*-R	GGAGCGTAGGCGGTTTTAC
*Selenomonas ruminantium*-F	CAATAAGCATTCCGCCTGGG
*Selenomonas ruminantium*-R	TTCACTCAATGTCAAGCCCTGG
*Ruminobacter amylophilus*-F	CTGGGGAGCTGCCTGAAT
*Ruminobacter amylophilus*-R	CATCTGAATGCGACTGGTTG
*Prevotella ruminicola*-F	GAAAGTCGGATTAATGCTCTATGTTG
*Prevotella ruminicola*-R	CATCCTATAGCGGTAAACCTTTGG

Abbreviations: F, forward primer; R, reverse primer.

**Table 3:** Chemical composition of fresh alfalfa.

Cultivar	DM, %	CP, % DM	EE, % DM	NDF, % DM	ADF, % DM
Fado	24.52 ± 0.89	23.42 ± 1.03 ^a^	4.02 ± 0.16 ^a^	37.35 ± 1.25	26.91 ± 1.15 ^d^
Instinct	25.44 ± 1.32	23.27 ± 1.61 ^a^	4.03 ± 0.13 ^a^	37.77 ± 1.15	26.93 ± 0.49 ^d^
Mezzo	25.28 ± 0.77	23.19 ± 0.82 ^a^	4.02 ± 0.10 ^a^	37.53 ± 0.83	26.63 ± 0.70 ^d^
Salsa	26.17 ± 1.05	23.08 ± 1.27 ^a^	4.01 ± 0.22 ^a^	37.23 ± 0.60	27.17 ± 0.13 ^cd^
Sibemol	24.09 ± 1.63	23.55 ± 1.88 ^a^	3.97 ± 0.24 ^a^	37.07 ± 0.68	26.99 ± 0.78 ^d^
Vision	25.98 ± 1.41	23.24 ± 1.69 ^a^	3.94 ± 0.18 ^a^	37.12 ± 0.75	26.86 ± 0.39 ^d^
Paola	25.87 ± 0.53	22.30 ± 0.53 ^ab^	3.85 ± 0.37 ^ab^	37.90 ± 0.56	27.93 ± 0.40 ^bcd^
MS-5S43	24.73 ± 0.81	22.64 ± 0.57 ^ab^	3.83 ± 0.17 ^ab^	38.02 ± 0.63	27.78 ± 0.32 ^bcd^
Power 5020	25.62 ± 0.92	22.15 ± 0.93 ^ab^	3.80 ± 0.20 ^ab^	38.04 ± 0.81	27.92 ± 0.05 ^bcd^
Sandili	25.51 ± 1.27	22.45 ± 1.15 ^bc^	3.83 ± 0.33 ^ab^	37.95 ± 0.25	27.97 ± 0.02 ^bcd^
Sidili	25.39 ± 0.62	22.26 ± 0.52 ^bc^	3.82 ± 0.20 ^ab^	37.92 ± 1.13	27.85 ± 0.59 ^bcd^
Villiger	24.25 ± 0.88	22.46 ± 0.86 ^bc^	3.83 ± 0.24 ^ab^	37.81 ± 0.95	27.82 ± 0.36 ^bcd^
Aurora	26.14 ± 1.18	21.24 ± 1.32 ^cd^	3.76 ± 0.15 ^bc^	38.27 ± 0.65	28.59 ± 0.21 ^abc^
Level 6	24.96 ± 0.71	21.63 ± 0.63 ^cd^	3.77 ± 0.07 ^bc^	38.39 ± 0.69	28.51 ± 0.07 ^abc^
Blue moon	24.82 ± 1.35	20.37 ± 1.42 ^cd^	3.67 ± 0.17 ^bcd^	38.73 ± 0.37	28.99 ± 0.63 ^ab^
Claudia	25.67 ± 1.16	20.62 ± 1.35 ^de^	3.68 ± 0.16 ^bcd^	38.79 ± 0.40	29.01 ± 0.36 ^ab^
Gannong No.5	25.53 ± 0.84	20.29 ± 0.97 ^de^	3.67 ± 0.17 ^bcd^	39.00 ± 0.62	28.78 ± 0.08 ^abc^
Sardi 7II	26.38 ± 1.57	20.70 ± 1.52 ^de^	3.71 ± 0.22 ^bcd^	38.82 ± 0.46	29.04 ± 0.11 ^ab^
Eureka+	25.24 ± 0.73	19.69 ± 0.68 ^ef^	3.64 ± 0.13 ^cd^	39.17 ± 0.52	29.26 ± 0.07 ^ab^
Pegasis	25.09 ± 1.39	18.91 ± 1.31 ^f^	3.57 ± 0.20 ^d^	39.46 ± 0.58	29.56 ± 0.12 ^a^
Sardi 10	24.95 ± 1.12	18.06 ± 1.05 ^f^	3.40 ± 0.39 ^d^	39.99 ± 0.78	30.00 ± 0.20 ^a^

Different lowercase letters indicate statistically significant differences (*p* < 0.05, one-way ANOVA; Tukey’s HSD). Data are presented as a mean ± SD (*n* = 4). Abbreviations: DM, dry matter; CP, crude protein; EE, ether extract; NDF, neutral detergent fiber; ADF, acid detergent fiber.

**Table 4:** Chemical composition of silage alfalfa.

Cultivar	DM, %	CP, % DM	EE, % DM	NDF, % DM	ADF, % DM	pH	Ammonia-N, % of Total N	Lactic Acid, % DM	Acetic Acid, % DM
Fado	39.04 ± 1.78	23.53 ± 1.11 ^a^	3.87 ± 0.28 ^ab^	33.35 ± 1.20	23.40 ± 1.35 ^bc^	4.67 ± 0.03	2.77 ± 0.37	5.89 ± 0.18 ^ab^	1.55 ± 0.09 ^a^
Instinct	40.88 ± 2.64	23.33 ± 1.75 ^ab^	3.99 ± 0.38 ^a^	33.71 ± 1.33	23.42 ± 0.69 ^bc^	4.65 ± 0.04	2.81 ± 0.13	5.88 ± 0.12 ^ab^	1.53 ± 0.10 ^a^
Mezzo	40.56 ± 1.54	23.37 ± 0.69 ^ab^	3.75 ± 0.19 ^abc^	33.50 ± 0.96	23.16 ± 1.00 ^c^	4.63 ± 0.12	2.79 ± 0.24	5.89 ± 0.20 ^ab^	1.54 ± 0.09 ^a^
Salsa	42.34 ± 2.10	23.25 ± 1.19 ^ab^	3.99 ± 0.15 ^a^	33.24 ± 0.70	23.63 ± 0.18 ^bc^	4.68 ± 0.06	2.78 ± 0.20	5.89 ± 0.10 ^ab^	1.55 ± 0.09 ^a^
Sibemol	38.18 ± 3.26	23.57 ± 1.94 ^a^	3.67 ± 0.13 ^abc^	33.10 ± 0.95	23.47 ± 1.09 ^bc^	4.72 ± 0.22	2.77 ± 0.18	5.88 ± 0.09 ^ab^	1.55 ± 0.02 ^a^
Vision	41.96 ± 2.82	23.41 ± 1.76 ^ab^	3.79 ± 0.33 ^abc^	33.15 ± 1.05	23.36 ± 0.69 ^c^	4.71 ± 0.19	2.77 ± 0.35	5.90 ± 0.07 ^a^	1.54 ± 0.06 ^a^
Paola	41.74 ± 1.06	22.34 ± 0.44 ^abc^	3.59 ± 0.18 ^bcd^	33.83 ± 0.78	24.29 ± 0.70 ^abc^	4.70 ± 0.05	2.66 ± 0.01	5.66 ± 0.15 ^abc^	1.45 ± 0.07 ^ab^
MS-5 S43	39.46 ± 1.62	22.54 ± 0.68 ^abc^	3.53 ± 0.11 ^bcd^	33.93 ± 0.88	24.16 ± 0.57 ^abc^	4.67 ± 0.12	2.63 ± 0.04	5.62 ± 0.10 ^abc^	1.45 ± 0.16 ^ab^
Power 5020	41.24 ± 1.84	22.25 ± 0.85 ^abc^	3.55 ± 0.10 ^bcd^	33.95 ± 1.13	24.28 ± 0.09 ^abc^	4.69 ± 0.05	2.68 ± 0.12	5.64 ± 0.19 ^abc^	1.44 ± 0.14 ^ab^
Sandili	41.02 ± 2.54	22.75 ± 1.13 ^ab^	3.65 ± 0.21 ^abc^	33.87 ± 0.35	24.32 ± 0.03 ^abc^	4.68 ± 0.06	2.66 ± 0.27	5.68 ± 0.19 ^abc^	1.45 ± 0.04 ^ab^
Sidili	40.78 ± 1.24	22.46 ± 0.45 ^abc^	3.51 ± 0.03 ^bcd^	33.84 ± 1.88	24.22 ± 1.06 ^abc^	4.71 ± 0.20	2.65 ± 0.37	5.66 ± 0.16 ^abc^	1.45 ± 0.03 ^ab^
Villiger	38.50 ± 1.76	22.54 ± 0.99 ^abc^	3.53 ± 0.08 ^bcd^	33.75 ± 1.60	24.19 ± 0.64 ^abc^	4.69 ± 0.06	2.66 ± 0.13	5.64 ± 0.12 ^abc^	1.44 ± 0.07 ^ab^
Aurora	42.28 ± 2.36	21.36 ± 1.24 ^bcd^	3.48 ± 0.27 ^bcd^	34.15 ± 0.90	24.86 ± 0.36 ^abc^	4.71 ± 0.03	2.63 ± 0.20	5.50 ± 0.02 ^bcd^	1.43 ± 0.02 ^ab^
Level 6	39.92 ± 1.42	21.53 ± 0.55 ^bcd^	3.45 ± 0.44 ^bcd^	34.25 ± 0.96	24.79 ± 0.13 ^abc^	4.69 ± 0.15	2.62 ± 0.07	5.51 ± 0.01 ^bcd^	1.42 ± 0.09 ^ab^
Blue moon	39.64 ± 2.70	20.56 ± 1.21 ^bcd^	3.37 ± 0.21 ^bcd^	34.55 ± 0.52	25.21 ± 1.12 ^abc^	4.68 ± 0.08	2.60 ± 0.13	5.40 ± 0.14 ^cd^	1.36 ± 0.08 ^ab^
Claudia	41.34 ± 2.32	20.77 ± 1.24 ^bcd^	3.40 ± 0.16 ^bcd^	34.60 ± 0.56	25.23 ± 0.64 ^abc^	4.63 ± 0.12	2.61 ± 0.06	5.42 ± 0.12 ^cd^	1.36 ± 0.10 ^ab^
Gannong No.5	41.06 ± 1.68	20.41 ± 0.91 ^bcd^	3.37 ± 0.36 ^bcd^	34.78 ± 1.03	25.03 ± 0.14 ^abc^	4.63 ± 0.13	2.60 ± 0.13	5.46 ± 0.08 ^bcd^	1.35 ± 0.04 ^ab^
Sardi 7II	42.76 ± 3.14	20.68 ± 0.44 ^bcd^	3.31 ± 0.16 ^bcd^	34.63 ± 0.78	25.25 ± 0.19 ^abc^	4.69 ± 0.06	2.59 ± 0.11	5.48 ± 0.15 ^bcd^	1.35 ± 0.03 ^ab^
Eureka+	40.48 ± 1.46	19.74 ± 0.62 ^cd^	3.15 ± 0.27 ^bcd^	34.93 ± 0.88	25.44 ± 0.12 ^abc^	4.71 ± 0.13	2.57 ± 0.22	5.29 ± 0.04 ^cd^	1.29 ± 0.02 ^ab^
Pegasis	40.18 ± 2.78	18.97 ± 0.25 ^cd^	3.06 ± 0.20 ^cd^	35.18 ± 0.98	25.70 ± 0.21 ^ab^	4.72 ± 0.06	2.55 ± 0.08	5.12 ± 0.07 ^d^	1.21 ± 0.05 ^b^
Sardi 10	39.90 ± 2.24	18.14 ± 1.00 ^d^	2.96 ± 0.09 ^d^	35.65 ± 1.30	26.09 ± 0.35 ^a^	4.76 ± 0.03	2.55 ± 0.09	5.10 ± 0.08 ^d^	1.22 ± 0.07 ^b^

Different lowercase letters indicate statistically significant differences (*p* < 0.05, one-way ANOVA; Tukey’s HSD). Data are presented as a mean ± SD (*n* = 4). Abbreviations: DM, dry matter; CP, crude protein; EE, ether extract; NDF, neutral detergent fiber; ADF, acid detergent fiber.

**Table 5:** Effects of the AS on the profile of VFA (volatile fatty acid).

Item	Control	FPS	SPS	FSS	SSS	FPES	SPES	FSES	SSES
Total VFA, mM	78.39 ± 0.47 ^d^	84.44 ± 1.00 ^c^	87.28 ± 0.56 ^b^	86.45 ± 0.75 ^c^	87.98 ± 0.74 ^b^	89.56 ± 0.20 ^a^	89.61 ± 0.30 ^a^	89.24 ± 0.09 ^a^	89.39 ± 0.14 ^a^
Acetate, %	68.32 ± 0.49 ^a^	65.98 ± 0.33 ^b^	64.88 ± 0.26 ^c^	65.89 ± 0.65 ^b^	64.51 ± 1.02 ^c^	61.59 ± 0.54 ^d^	61.25 ± 0.56 ^d^	61.69 ± 0.07 ^d^	61.56 ± 0.46 ^d^
Propionate, %	17.99 ± 0.57 ^d^	20.71 ± 0.61 ^c^	21.66 ± 0.70 ^b^	21.20 ± 0.77 ^c^	21.94 ± 0.58 ^b^	24.10 ± 0.28 ^a^	23.84 ± 0.48 ^a^	23.65 ± 0.64 ^a^	23.55 ± 0.52 ^a^
Butyrate, %	7.51 ± 0.39 ^c^	7.05 ± 0.18 ^d^	7.32 ± 0.21 ^c^	6.84 ± 0.83 ^d^	7.37 ± 0.56 ^c^	8.57 ± 0.19 ^a^	8.70 ± 0.27 ^a^	8.68 ± 0.28 ^a^	8.77 ± 0.61 ^a^
Iso-butyrate, %	2.08 ± 0.05 ^a^	1.60 ± 0.05 ^c^	1.57 ± 0.02 ^c^	1.64 ± 0.04 ^c^	1.57 ± 0.05 ^c^	1.93 ± 0.03 ^a^	2.05 ± 0.08 ^a^	1.90 ± 0.07 ^b^	1.93 ± 0.06 ^b^
Valerate, %	1.74 ± 0.03	1.53 ± 0.02	1.50 ± 0.03	1.53 ± 0.03	1.51 ± 0.05	1.51 ± 0.03	1.60 ± 0.05	1.53 ± 0.03	1.65 ± 0.02
Iso-valerate, %	2.36 ± 0.32	3.13 ± 0.36	3.07 ± 0.27	2.91 ± 0.49	3.11 ± 0.44	2.29 ± 0.26	2.55 ± 0.17	2.56 ± 0.40	2.54 ± 0.21
Acetate to propionate ratio	3.80 ± 0.09 ^a^	3.19 ± 0.11 ^b^	3.00 ± 0.11 ^c^	3.11 ± 0.09 ^b^	2.95 ± 0.12 ^c^	2.56 ± 0.05 ^d^	2.57 ± 0.07 ^d^	2.61 ± 0.07 ^d^	2.62 ± 0.05 ^d^

Values represent the molar proportion of individual VFA as a percentage of total VFA. VFA concentrations were log-transformed before analysis to meet normality assumptions (Shapiro–Wilk *p* > 0.10). Different lowercase letters indicate statistically significant differences (*p* < 0.05, one-way ANOVA; Tukey’s HSD). Data are presented as a mean ± SD (*n* = 4). Abbreviations: FPS, fresh Pegasis saponin; SPS, silage Pegasis saponin; FSS, fresh Salsa saponin; SSS, silage Salsa saponin; FPES, fresh Pegasis enzymatic saponin; SPES, silage Pegasis enzymatic saponin; FSES, fresh Salsa enzymatic saponin; SSES, silage Salsa enzymatic saponin.

## Data Availability

Data are contained within the article and will be available upon request.
